# A systematic scoping review of reflective writing in medical education

**DOI:** 10.1186/s12909-022-03924-4

**Published:** 2023-01-09

**Authors:** Jia Yin Lim, Simon Yew Kuang Ong, Chester Yan Hao Ng, Karis Li En Chan, Song Yi Elizabeth Anne Wu, Wei Zheng So, Glenn Jin Chong Tey, Yun Xiu Lam, Nicholas Lu Xin Gao, Yun Xue Lim, Ryan Yong Kiat Tay, Ian Tze Yong Leong, Nur Diana Abdul Rahman, Min Chiam, Crystal Lim, Gillian Li Gek Phua, Vengadasalam Murugam, Eng Koon Ong, Lalit Kumar Radha Krishna

**Affiliations:** 1 Yong Loo Lin School of Medicine, National University of Singapore, NUHS Tower Block, 1E Kent Ridge Road, Level 11, Singapore, 119228 Singapore; 2 Division of Supportive and Palliative Care, National Cancer Centre Singapore, 11 Hospital Crescent, Singapore, 169610 Singapore; 3 Division of Medical Oncology, National Cancer Centre Singapore, 11 Hospital Crescent, Singapore, 169610 Singapore; 4 Division of Cancer Education, National Cancer Centre Singapore, 11 Hospital Crescent, Singapore, 169610 Singapore; 5 Duke-NUS Medical School, National University of Singapore, 8 College Rd, Singapore, 169857 Singapore; 6 Medical Social Services, Singapore General Hospital, Outram Rd, Singapore, 169608 Singapore; 7 Lien Centre for Palliative Care, Duke-NUS Medical School, National University of Singapore, 8 College Rd, Singapore, 169857 Singapore; 8 Assisi Hospice, 832 Thomson Rd, Singapore, 574627 Singapore; 9 Palliative Care Institute Liverpool, Academic Palliative & End of Life Care Centre, Cancer Research Centre, University of Liverpool, 200 London Road, Liverpool, L3 9TA UK; 10 PalC, The Palliative Care Centre for Excellence in Research and Education, PalC c/o Dover Park Hospice, 10 Jalan Tan Tock Seng, Singapore, 308436 Singapore

**Keywords:** Reflection, Reflective writing, Medical education, Professional identity formation, Undergraduate medical education, Postgraduate medical education

## Abstract

**Background:**

Reflective writing (RW) allows physicians to step back, review their thoughts, goals and actions and recognise how their perspectives, motives and emotions impact their conduct. RW also helps physicians consolidate their learning and boosts their professional and personal development. In the absence of a consistent approach and amidst growing threats to RW’s place in medical training, a review of theories of RW in medical education and a review to map regnant practices, programs and assessment methods are proposed.

**Methods:**

A Systematic Evidence-Based Approach guided Systematic Scoping Review (SSR in SEBA) was adopted to guide and structure the two concurrent reviews. Independent searches were carried out on publications featured between 1st January 2000 and 30th June 2022 in PubMed, Embase, PsychINFO, CINAHL, ERIC, ASSIA, Scopus, Google Scholar, OpenGrey, GreyLit and ProQuest. The Split Approach saw the included articles analysed separately using thematic and content analysis. Like pieces of a jigsaw puzzle, the Jigsaw Perspective combined the themes and categories identified from both reviews. The Funnelling Process saw the themes/categories created compared with the tabulated summaries. The final domains which emerged structured the discussion that followed.

**Results:**

A total of 33,076 abstracts were reviewed, 1826 full-text articles were appraised and 199 articles were included and analysed. The domains identified were theories and models, current methods, benefits and shortcomings, and recommendations.

**Conclusions:**

This SSR in SEBA suggests that a structured approach to RW shapes the physician’s belief system, guides their practice and nurtures their professional identity formation. In advancing a theoretical concept of RW, this SSR in SEBA proffers new insight into the process of RW, and the need for longitudinal, personalised feedback and support.

**Supplementary Information:**

The online version contains supplementary material available at 10.1186/s12909-022-03924-4.

## Introduction

Reflective practice in medicine allows physicians to step back, review their actions and recognise how their thoughts, feelings and emotions affect their decision-making, clinical reasoning and professionalism [1]. This approach builds on Dewey [2], Schon [3, 4], Kolb [5], Boud et al. [6] and Mezirow [7]’s concepts of critical self-examination. It sees new insights drawn from the physician’s experiences and considers how assumptions may integrate into their current values, beliefs and principles (henceforth belief system) [8, 9].

Teo et al. [10] build on this concept of reflective practice. The authors suggest that the physician’s belief system informs and is informed by their self-concepts of identity which are in turn rooted in their self-concepts of personhood - how they conceive what makes them who they are [11]. This posit not only ties reflective practice to the shaping of the physician’s moral and ethical compass but also offers evidence of it's role in their professional identity formation (PIF) [8, 12–23]. With PIF [8, 24] occupying a central role in medical education, these ties underscore the critical importance placed on integrating reflective practice in medical training.

Perhaps the most common form of reflective practice in medical education is reflective writing (RW) [25]. Identified as one of the distinct approaches used to achieve integrated learning, education, curriculum and teaching [26], RW already occupies a central role in guiding and supporting longitudinal professional development [27–29]. Its ability to enhance self-monitoring and self-regulation of decisional paradigms and conduct has earned RW a key role in competency-based medical practice and continuing professional development [30–36].

However, the absence of consistent guiding principles, dissonant practices, variable structuring and inadequate assessments have raised concerns as to RW’s efficacy and place in medical training [25, 37–39]. A Systematic Scoping Review is proposed to map current understanding of RW programs. It is hoped that this SSR will also identify gaps in knowledge and regnant practices, programs and assessment methods to guide the design of RW programs.

## Methodology

A Systematic Scoping Review (SSR) is employed to map the employ, structuring and assessment of RW in medical education. An SSR-based review is especially useful in attending to qualitative data that does not lend itself to statistical pooling [40–42] whilst its broad flexible approach allows the identification of patterns, relationships and disagreements [43] across a wide range of study formats and settings [44, 45].

To synthesise a coherent narrative from the multiple accounts of reflective writing, we adopt Krishna’s Systematic Evidence-Based Approach (SEBA) [10, 15, 21, 46–53]. A SEBA-guided Systematic Scoping Review (SSR in SEBA) [13–24, 50, 53–55] facilitates reproducible, accountable and transparent analysis of patterns, relationships and disagreements from multiple angles [56].

**Fig. 1:**
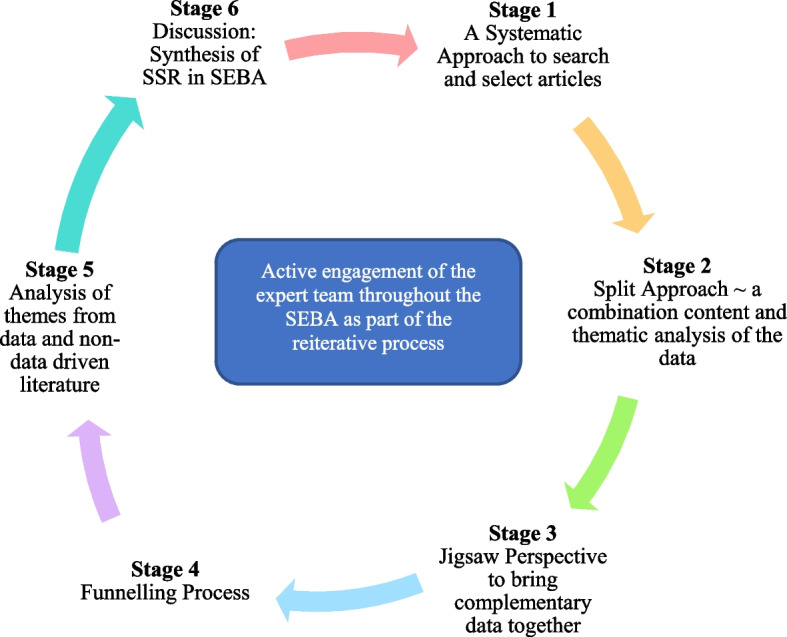
The SEBA Process

The SEBA process (Fig. 1) comprises the following elements: 1) Systematic Approach, 2) Split Approach, 3) Jigsaw Perspective, 4) Funnelling Process, 5) Analysis of data and non-data driven literature, and 6) Synthesis of SSR in SEBA [10, 15, 21, 46–53, 57–60] . Every stage was overseen by a team of experts that included medical librarians from the Yong Loo Lin School of Medicine (YLLSoM) at the National University of Singapore, and local educational experts and clinicians at YLLSoM, Duke-NUS Medical School, Assisi Hospice, Singapore General Hospital, National Cancer Centre Singapore and Palliative Care Institute Liverpool.

### STAGE 1 of SEBA: Systematic Approach

#### Determining the title and background of the review

Ensuring a systematic approach, the expert team and the research team agreed upon the overall goals of the review. Two separate searches were performed, one to look at the theories of reflection in medical education, and another to review regnant practices, programs, and assessment methods used in reflective writing in medical education. The PICOs is featured in Table 1.

**Table 1:** PICOs inclusion and exclusion criteria

	Inclusion criteria	Exclusion criteria
**Search #1: Theories of reflection in medical education**		
Population	• Healthcare personnel and educators in allied health specialities and medicine • Undergraduate and postgraduate medical students • Physicians	• Non-healthcare educators and specialities
Intervention	• Papers addressing theory building relevant to reflection or reflective practices in education	• Evaluation of reflective practices without reference to theory relating to reflection or reflective practices in education • Evaluation of reflective practices for purposes other than improving reflective capacity of users • Papers with little detail on implementation or assessment details of reflective writing
Comparison Outcome	• Comparison of various modes of reflective practices and how they differed in terms of theory • Impact of the use of reflective writing within the clinical, medical, research and/or academic settings Papers that discussed reflective writing without the above comparisons were also included	
Study design	• All study designs including mixed methods research, meta-analyses, systematic reviews, randomised controlled trials, cohort studies, case-control studies, cross-sectional studies, descriptive papers, grey literature, opinions, letters, commentaries and editorials • Articles in English or translated to English • Year of Publication: Jan 2000–Jun 2022	• Non-English language articles
**Search #2: Reflective writing in medical education**		
Population	• Junior doctors, residents, specialists and/or doctors and/or physicians and/or medical students within the clinical, medical, research and/or academic settings • Undergraduate and postgraduate medical students	• Allied health specialties such as Pharmacy, Dietetics, Chiropractic, Midwifery, Podiatry, Speech Therapy, Occupational and Physiotherapy, Physician Assistants • Non-medical specialties such as Clinical and Translational Science, Alternative and Traditional Medicine, Veterinary, Dentistry
Intervention	• Papers that addressed the incorporation of reflective writing for junior doctors, residents, specialists and/or doctors and/or physicians and/or medical students within the clinical, medical, research and/or academic settings • Papers that addressed assessment of reflective writing	• Papers with little detail of implementation or assessment of reflective writing in curriculum • Papers that evaluated reflective writing for purposes other than improving reflective capacity of users
Comparison Outcome	Papers that addressed the following comparisons were also included: • Comparison of the various uses of reflective writing in different teaching settings • Evaluation of the effectiveness of reflective writing in comparison to other educational interventions • Papers that discussed reflective writing without the above comparisons were also included Papers that measured the following outcomes were also included: • Impact of the use of reflective writing on junior doctors, residents, specialists and/or doctors and/or physicians and/or medical students within the clinical, medical, research and/or academic settings • Impact of the use of reflective writing on teaching • Impact of the use of reflective writing on assessment • Gaps and improvements to current reflective writing programs	
Study design	• All study designs including: mixed methods research, meta-analyses, systematic reviews, randomised controlled trials, cohort studies, case-control studies, cross-sectional studies, descriptive papers, grey literature, opinions, letters, commentaries and editorials • Articles in English or translated to English • Year of Publication: Jan 2000–Jun 2022	• Non-English language articles

#### Identifying the research question

Guided by the Population Concept, Context (PCC) elements of the inclusion criteria and through discussions with the expert team, the research question was determined to be: “*How is reflective writing structured, assessed and supported in medical education?*” The secondary research question was “*How might a reflective writing program in medical education be structured?*”

#### Inclusion criteria

All study designs including grey literature published between 1st January 2000 to 30th June 2022 were included [61, 62]. We also consider data on medical students and physicians from all levels of training (henceforth broadly termed as physicians).

#### Searching

Ten members of the research team carried out independent searches using seven bibliographic databases (PubMed, Embase, PsychINFO, CINAHL, ERIC, ASSIA, Scopus) and four grey literature databases (Google Scholar, OpenGrey, GreyLit, ProQuest). Variations of the terms “reflective writing”, “physicians and medical students”, and “medical education” were applied.

#### Extracting and charting

Titles and abstracts were independently reviewed by the research team to identify relevant articles that met the inclusion criteria set out in Table 1. Full-text articles were then filtered and proposed. These lists were discussed at online reviewer meetings and Sandelowski and Barroso [63]’s approach to ‘negotiated consensual validation’ was used to achieve consensus on the final list of articles to be included.

### Stage 2 of SEBA: Split Approach

The Split Approach was employed to enhance the trustworthiness of the SSR in SEBA [64, 65]. Data from both searches were analysed by three independent groups of study team members.

The first group used Braun and Clarke [66]’s approach to thematic analysis. Phase 1 consisted of ‘actively’ reading the included articles to find meaning and patterns in the data. The analysis then moved to Phase 2 where codes were constructed. These codes were collated into a codebook and analysed using an iterative step-by-step process. As new codes emerge, previous codes and concepts were incorporated. In Phase 3, codes and subthemes were organised into themes that best represented the dataset. An inductive approach allowed themes to be “defined from the raw data without any predetermined classification” [67]. In Phase 4, these themes were then further refined to best depict the whole dataset. In Phase 5, the research team discussed the results and consensus was reached, giving rise to the final themes.

The second group employed Hsieh and Shannon [68]’s approach to directed content analysis. Categories were drawn from Mann et al. [9]’s article, *“Reflection and Reflective Practice in Health Professions Education: A Systematic Review”* and Wald and Reis [69]’s article *“Beyond the Margins: Reflective Writing and Development of Reflective Capacity in Medical Education”.*

The third group created tabulated summaries in keeping with recommendations drawn from Wong et al. [56]’s *"RAMESES Publication Standards: Meta-narrative Reviews"* and Popay et al. [70]’s *“Guidance on the C*onduct of *N*arrative Synthesis in Systematic *Reviews”.* The tabulated summaries served to ensure that key aspects of included articles were not lost.

### Stage 3 of SEBA: Jigsaw Perspective

The Jigsaw Perspective [71, 72] saw the findings of both searches combined. Here, overlaps and similarities between the themes and categories from the two searches were combined to create themes/categories. The themes and subthemes were compared with the categories and subcategories identified, and similarities were verified by comparing the codes contained within them. Individual subthemes and subcategories were combined if they were complementary in nature.

### Stage 4 of SEBA: Funnelling Process

The Funnelling Process saw the themes/categories compared with the tabulated summaries to determine the consistency of the domains created, forming the basis of the discussion.

### Stage 5: Analysis of data and non-data driven literature

Amidst concerns that data from grey literature which were neither peer-reviewed nor necessarily evidence-based may bias the synthesis of the discussion, the research team separately thematically analysed the included grey literature. These themes were compared with themes from data-driven or research-based peer-reviewed data and were found to be the same and thus unlikely to have influenced the analysis.

### Stage 6: Synthesis of SSR in SEBA

The Best Evidence Medical Education (BEME) Collaboration Guide and the Structured approach to the Reporting In healthcare education of Evidence Synthesis (STORIES) were used to guide the discussion.

## Results

**Fig. 2:**
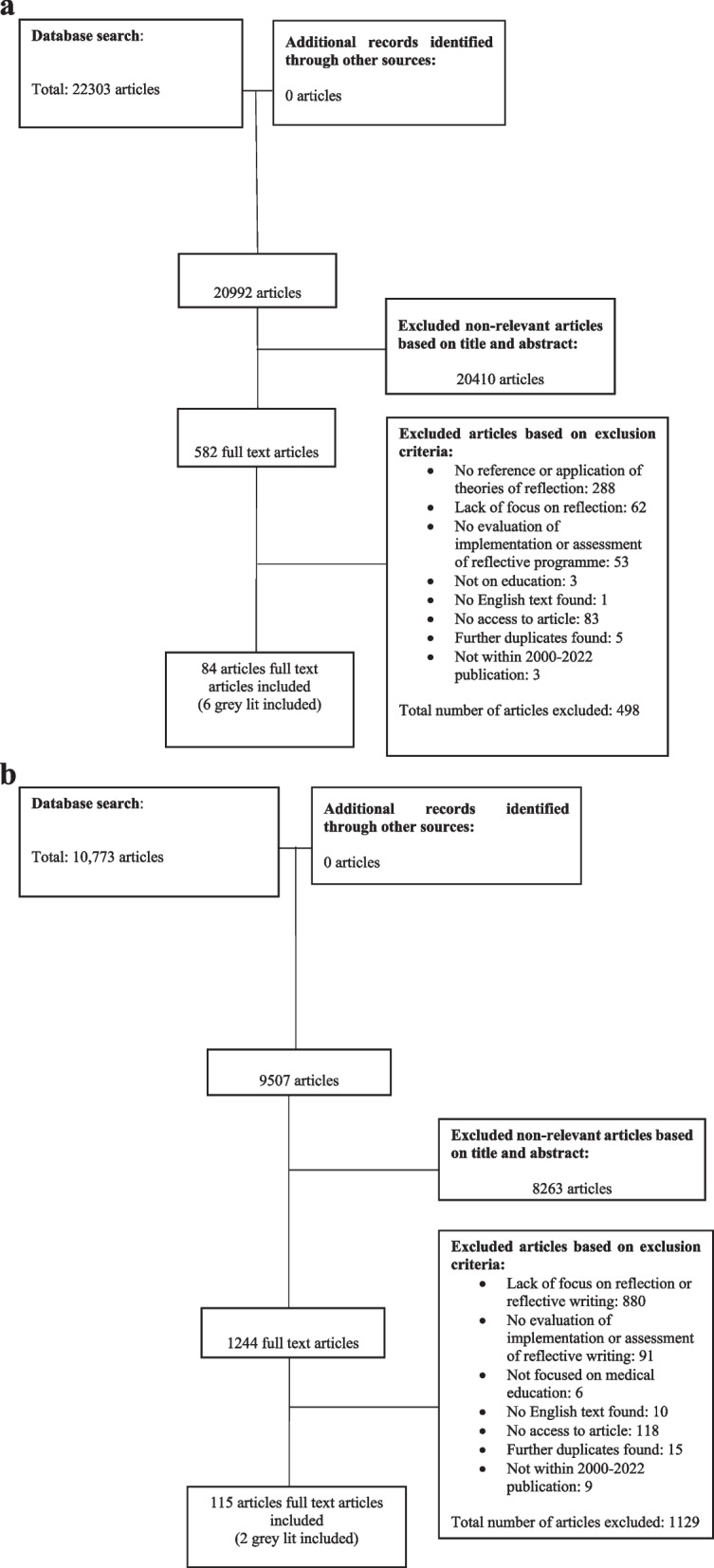
**a** PRISMA Flow Chart (Search Strat #1: Theories of Reflection in Medical Education). **b** PRISMA Flow Chart (Search Strat #2: Reflective Writing in Medical Education)

A total of 33,076 abstracts were reviewed from the two separate searches on theories of reflection in medical education, and on regnant practices, programs and assessments of RW programs in medical education. A total of 1826 full-text articles were appraised from the separate searches, and 199 articles were included and analysed. The PRISMA Flow Chart may be found in Fig. 2a and b. The domains identified when combining the findings of the two separate searches were 1) Theories and Models, 2) Current Methods, 3) Benefits and Shortcomings and 4) Recommendations.

### Domain 1: Theories and Models

Many current theories and models surrounding RW in medical education are inspired by Kolb’s Learning Cycle [5] (Table 2). These theories focus on descriptions of areas of reflection; evaluations of experiences and emotions; how events may be related to previous experiences; knowledge critiques of their impact on thinking and practice; integration of learning points; and the physician’s willingness to apply lessons learnt [6, 73–75]. In addition, some of these theories also consider the physician’s self-awareness, ability and willingness to reflect [76], contextual factors related to the area of reflection [4, 77] and the opportunity to reflect effectively within a supportive environment [78, 79]. Ash and Clayton's DEAL Model recommends inclusion of information from all five senses [80–83]. Johns's Model of Structured Reflection [84] advocates giving due consideration to internal and external influences upon the event being evaluated. Rodgers [39] underlines the need for appraisal of the suppositions and assumptions that precipitate and accompany the effects and responses that may have followed the studied event. Griffiths and Tann [75], Mezirow [77], Kim [85], Roskos et al. [86], Burnham et al. [87], Korthagen and Vasalos [78] and Koole et al. [74] build on Dewey [2] and Kolb [5]’s notion of creating and experimenting with a ‘working hypothesis’. These models also propose that the lessons learnt from experimentations should be critiqued as part of a reiterative process within the reflective cycle. Underlining the notion of the reflective cycle and the long-term effects of RW, Pearson and Smith [88] suggest that reflections should be carried out regularly to encourage longitudinal and holistic reflections on all aspects of the physician’s personal and professional life.

**Table 2:** Theories and models referred for implementation - iterative stages of reflection

Author	Process of reflection			
	***Description of event***	***Deconstructing event***	***Learning outcomes***	*Framing*
Schon's Reflection-in-Action and Reflection-on-Action [4]	Knowing in action, Reflection-in-action, Reflection-on-action			
Argyris and Schon's Organisational Learning [89]	Single and Double loop learning			
Gibbs' Reflective Cycle [73]	Description *What happened?*	Evaluation *What was good and bad about the experience?*	Action Plan *If it arose again, what would you do?*	
	Feelings *What were you thinking and feeling?*	Analysis *What else can you make of the situation?*		
		Conclusion *What else could you have done?*		
Kolb’s Learning Cycle [5]	Concrete experience *Doing/ having an experience*	Reflective observation *Reviewing/ reflecting on experience*	Abstract conceptualisation *Concluding/ learning from experience*	
			Active experimentation *Planning/ trying out what you have learned*	
Kim’s Critical Reflective Inquiry [85]	Description of situation	Reflection and analysis of situation	Critical phase focused on correcting ineffective practice and moving to changed perspectives and actions	
Boud's Reflection Model [6]	Experience *Behaviour* *Ideas* *Feelings*	Reflective process *Returning to experience* *Attending to feelings* *Re-evaluating experience*	Outcomes *New perspectives* *Change in behaviour* *Readiness for application* *Commitment to action*	
Griffiths and Tann's 5 Level Model of Reflection [75]	Action *Rapid reaction (immediate)*	Analysis *Review (after the event)*	Planning *Retheorize/ reformulate (formal and rigorous appraisal)*	
	Observation *Repair (momentary)*	Evaluation *Research (systematic)*		
Mamede and Schmidt's 5-Factor Model of Reflective Practice [90]	Reporting *What were your feelings and responses to the situation?*	Relating *Are there any connections between this event and your past experience and understanding?*	Reconstructing *In the future, can you develop some action plans based on this event?*	
		Reasoning *Can you analyse more about the event? Did you find any significant factors underlying this clinical encounter?*	Reflecting *Can you give some feedback on this debriefing?*	
Ryan’s 4Rs of Reflection [91]	Reporting *What happened?*	Relating *Have I seen this before?* *Were the conditions same or different?* *Do I have the skills or knowledge to deal with this?*	Reconstructing *How would I deal with this next time?* *What might work and why?* *What might happen if…?* *Are my ideas supported by theory?*	
		Reasoning *Factors underlying issue* *Why they are important* *Refer to relevant theory* *Consider different perspectives*		
Beauchamp's Integrative Framework [92]	Examining	Thinking and understanding	Developing and transforming	Concerning a particular object, and in view of achieving a particular goal, or rationale
		Problem solving		
		Analysing		
		Evaluating and/ or constructing		
Pearson and Smith's Debriefing [88]	Log *What happened?*	Diary *How do you feel?*	Journal *What does it all mean?*	
Johns' Model of Structured Reflection [84]	Description of experience *Phenomenon* *Describe the ‘here and now’ experience*	Reflection *What was I trying to achieve?* *Why did I intervene/ react as I did?* *What were the consequences of my actions?* *How did I feel about this experience?* *How did the other person feel? How do I know how the other person felt?*	Learning *What other choices did I have* *What would be the consequences* *how do i feel now* *how have i made sense of this experience* *how has this experience changed my ways of knowing*	
		Influencing factors *Internal factors* *External factors* *Sources of knowledge*		
Koole et al.'s 'Eclectic' Model [74]	Reviewing the experience *Adequate description of an event* *Identify essential elements and describe own thoughts and feelings*	Critical analyses *Searching questions* *Frames of reference*	Reflective outcome *Conclusions* *Concrete learning goals* *Plans for future actions*	
Dewey’s 5 Phases [2]	Disturbance and uncertainty	Studying conditions of situation and formation of working hypothesis	Testing hypothesis in action	
	Intellectualisation and definition of problem	Reasoning		
Atkins and Murphy's Model of Reflection [93]	Identify and learning	Analysis		
	Awareness of discomfort or action or experience	Evaluate		
	Describe the situation			
Roskos et al.'s Reflection and Learning [86]	Describe an activity	Interpret activity	Critique activity	
		Evaluate activity		
Mezirow’s Transformative Learning [77]	Disorienting dilemma	Critical assessment of assumptions	Exploration of new roles, relationships and actions	
	Self-examination with feelings	Recognition of one’s discontent	Planning a course of action	
			Acquiring knowledge and skills for implementing plans	
			Provisional trying of new roles	
			Building competence and self confidence in new roles	
			Reintegration of new perspectives	
Ash and Clayton's DEAL Model [80–83]	Describe *Factual overview* *5 senses*	Examine	Articulate learning *What was learned?* *How it was learned?* *Why is it important?* *How learning can be applied to future practices?*	
Korthagen's ALACT Model of Reflection [78]	Acting	Awareness of essential aspects	Creating alternative methods of action	
	Looking back on action		Trial	
McLeod's 9 Steps of Reflection [79]	Readiness to be open	Recognising personal influences	Responding by making appropriate changes	
	Recalling situation	Reflecting on experiences from other’s perspectives	Remembering benefits of learning	
		Reviewing		
		Relating to relevant reading		
		Re-appraising relevance		
Bass et al.'s Model of Holistic Reflection [76]	Self awareness	Reflection *Thoughts and feelings*	Learning *Synthesis/ action*	
	Description	Influences		
		Evaluation *Analysis/ conclusions*		
Carver and Scheier's Model of Behavioural Self-Regulation [94]				Context of goals wished to pursue
Grant's Life Coaching [95]				Context of goals wished to pursue
Burnham's GGRRAAACCEEESSS Model [87]				Gender, geography, race, religion, age, ability, appearance, class, culture, ethnicity, education, employment, sexuality, sexual orientation and spirituality

Regnant theories shape assessments of RW (Table 3). This extends beyond Thorpe [96]’s study which categorises reflective efforts into ‘non-reflectors’, ‘reflectors’, ‘critical reflectors’, and focuses on their process, structure, depth and content. van Manen [97], Plack et al. [98], Rogers et al. [99] and Makarem et al. [100] begin with evaluating the details of the events. Kim’s Critical Reflective Inquiry Model [85] and Bain’s 5Rs Reflective Framework [101] also consider characterisations of emotions involved. Other models appraise the intentions behind actions and thoughts [85], the factors precipitating the event [101] and meaning-making [85]. Other theories consider links with previous experiences [100], the integration of thoughts, justifications and perspectives [99], and the hypothesising of future strategies [98].

**Table 3:** Theories and models referred for assessment - vertical levels of reflection

Author	Depth of reflection			
	Non-reflectors (e.g. habitual reflection, thoughtful action, introspection)	Reflectors (e.g. content reflection, process reflection, content and process reflection)	Critical reflectors (e.g. Premise reflection)	Content of reflection/ criterion
Kember et al.'s Reflective Thinking Scale [102]	Habitual action, Understanding	Reflection	Critical reflection	
Hatton and Smith's 4 Levels of Reflective Writing [103]	Description	Descriptive reflection, Dialogic reflection	Critical reflection	
Dewey's 5 Phases [2]		Content and process reflection	Premise/ critical reflection	
Moon's Map of Learning [104]	Noticing, Making sense	Making meaning, Working with meaning	Transformative learning	
Mezirow's Transformative Learning [7]	Habitual action, Thoughtful action, Understanding	Reflection	Critical reflection	
Wald et al.'s REFLECT Rubric [105]	Habitual action Thoughtful action or introspection	Reflection	Critical reflection	Writing spectrum Presence/ sense of writer Description of conflict or disorienting dilemma Attending to emotions Analysis and meaning making
Stein's Critical Reflection [106]	No evidence of reflection (Descriptive only, no suggestions for maintaining strengths and improving weaknesses)	Developing reflection (Strengths and weaknesses identified; incorporation of two of following: patient feedback, past experience, evidence for patient-centered interviewing)	Deep reflection	Skills Feelings Rationale Patient’s reactions Patient feedback Patient-centered interviewing
Bain's 5Rs Reflective Framework [101]	Component 1: Reporting (Micro-reflection) i.e. *Describing what happened*	Component 3: Relating (Micro-reflection) i.e. *Finding connections between incident and writer’s own experiences and understanding*	Component 5: Reconstructing (Micro-reflection) i.e. *Reframing or reconstruction of future practices and own understanding*	
	Component 2: Responding (Micro-reflection) i.e. *Making observations, expressing feelings or asking questions*	Component 4: Reasoning (Micro-reflection) i.e. *Identifying factors underlying incident*	Component 6: Representing (Macro-reflection) i.e. *Framing of reflection into local, regional, national and global context*	
Morrow's Critical Reflection [107]				Personal Interpersonal Contextual Critical/ Evaluation – limitations faced, social, ethical problems faced
Plack et al.'s Method of Assessing Reflective Journal Writing [108]	No evidence of reflection	Evidence of reflection	Evidence of critical reflection i.e. exploration of existence of problem, where problems arises from, underlying assumptions; revisits experience to challenge assumptions and modification of biases	
Kims’s Critical Reflective Inquiry Model [85]	Descriptive *Description of practice events, actions, thoughts and feelings*	Reflective *Analysis of situation, of intentions*	Critical *Critique of practice regarding conflicts, distortion and inconsistencies* *Engagement in emancipatory change process*	
Makaram et al.'s GRE-9 [100]	*What happened?* *What is special about this event?* *Feelings when it happened?* *What was the outcome for the concerned?* *Understanding of the event*	Congruence of actions and beliefs New thoughts and feelings after reflection	Reference to old experience and others How this incident will affect future role	
Aukes et al.'s Groningen Reflection Ability Scale [109]				Self-reflection Empathetic reflection Reflective communication
Wang and Liao's Analytic Reflective Writing Scoring Rubric for Healthcare Students and Providers [1]				Focus and contextualisation Ideas and elaboration Voices and points of view Critical thinking and representation Depth of reflection regarding personal growth Language and style
Plack et al.'s Modified Cuppernull Bloom’s Taxonomy [98]	Level 1: Knowledge and comprehension *Description of event*	Level 2: Analysis *Deconstruction of experience, examination of alternative explanations*	Level 3: Synthesis and evaluation *Conclusions* *Hypothesize different strategies for future* *Articulation of learning*	
Rogers et al.'s Reflection Rubric [99]	Beginning i.e. *Thoughts conveyed but no to minimal integration of personal thoughts into experience/ justification/ based on one or two perspectives with no to minimal evidence*	Developing i.e. *some integration of personal thoughts/ some justification/ two perspectives with some evidence*	Distinguished i.e. *strong integration of personal thoughts/ substantial justification/ more than two perspectives with substantial evidence*	Presentation Perspective taking Connection Understanding-cognition Understanding-emotion
		Proficient i.e. *Moderate integration of personal thoughts/ moderate justification/ two perspectives with moderate evidence*		
Bradley's Model for Evaluating Student Learning [110]	Descriptive	Analytical	Integrative *Impact on global issues*	
Lee’s 3 Levels of Reflection [111]	Recall level (R1) *Description*	Rationalisation level (R2) *Reasons and rationale* *Guiding principles*	Reflectivity level (R3) *Perspective finding*	
van Manen's Tact of Teaching [97]	Technical rationality Practical action *Description of event*		Critical reflection *Using personal and other’s experiences to systematically examine phenomenon* Reflection on reflection *Metacognitive processing*	

### Domain 2: Current methods of structuring RW programs

Current programs focus on supporting the physician throughout the reflective process. Whilst due consideration is given to the physician’s motivations, insight, experiences, capacity and capabilities [25, 96, 112–116], programs also endeavour to ensure appropriate selection and training of physicians intending to participate in RW. Efforts are also made to align expectations, and guide and structure the RW process [37, 116–122]. Physicians are provided with frameworks [76, 79, 105, 123, 124], rubrics [99, 123, 125, 126], examples of the expected quality and form of reflection [96, 115, 116], and how to include emotional and contextual information in their responses [121, 127–129].

Other considerations are enclosed in Table 4 including frequency, modality and the manner in which RW is assessed.

**Table 4:** Current methods of structuring RW programs

Methods of structuring RW programs	Elaboration
Structured vs unstructured reflection	Orientation of user to benefits of reflection and key aspects of reflection [25, 96, 112–116] ° Novices requiring explicit instructions [130] ° Practice sessions for reflective journaling at the beginning of program [114] Prompt questions and suggested frameworks ° To recount and describe event [114, 121, 131, 132] ° To retrospectively analyse own behaviour and rationalise actions [114, 121, 131, 133–137] ° To reflect on emotions and feelings [121, 127–129] ° Action for learning [114, 121, 132–134, 136, 138, 139] ° No frameworks, structure or prompts given to users [120, 140, 141] Suggested events to reflect on ° On self-identified significant clinical encounters [37, 116–122] ° On competency domains [113, 119, 142, 143] ° On hypothetical scenarios [144] Examples of good reflection given to users [96, 115, 116] Benefits of scaffolding ° Frameworks help users to obtain greater breadth and depth in their reflective capacity [76, 79, 105, 123, 124] and can be used as an assessment rubric and guide for self-reflection processes [99, 123, 125, 126], especially for new users [138] ° Simple frameworks allow for RW to be assessed with limited faculty training time or high volume of written reflections to be scored [145] ° Ease of use allows users to peer assess one another [126] Cons of scaffolding ° Prompts could restrict ability of users to engage in reflective writing [146]
Frequency of reflection	Once-off [112, 115, 118, 123, 139, 142, 144, 145, 147–154] Thrice weekly [155, 156] Weekly [116, 122, 136, 157–164] Bi-weekly [117, 133, 165] Monthly [135] Daily [119, 134]
Modality of reflection	Modality of reflection ° Electronic portfolios ° Written reflective essays/ journals ° Oral narration (i.e. interviews, focused groups discussion) ° Written and verbal adjunct ° Written and video adjunct Comparison of e-journals with hardcopy journals ° Benefits of e-journals: convenience, ease of use, immediacy in terms of feedback, accessibility and visual impact [29, 162, 166] Use of video journals ° Allows for more authentic responses which can later be reviewed, discussed and reflected upon in sessions [167]
Group vs individual activity	Face to face meetings for feedback/ discussion ° One-on-one meetings [30, 119, 128, 143, 148, 150, 167, 168] ° Small group discussions [96, 115, 148, 169–175] Provision of feedback/ sharing of reflections ° Assurance of confidentiality [96, 120, 148, 152, 176, 177] ° Importance of feedback for improvement of experience [30, 96, 173, 178–180] ° Peer to peer feedback allowed for increased sense of camaraderie with classmates [120, 181] ° Peer to peer feedback allowed for enhanced learning [69], increased awareness of personal strengths, while self-reflection enhanced personal weaknesses [173] ° Peer to peer relationships oscillate between support and judgement [149]
Formative vs summative assessment	Formatives Summative No assessment given Dilemmas regarding assessment of RW ° Compulsory assessments encourage users to take assignments seriously and participate [114, 182] ° Assessments allow for developing of reflective skills [183] ° Compulsory assessments result in users writing down what they believe is expected of them instead of their own genuine responses [114, 143, 155, 184]

### Domain 3: Benefits and Shortcomings

The benefits of RW are rarely described in detail and may be divided into personal and professional benefits as summarised in Table 5 for ease of review. From a professional perspective, RW improves learning [96, 112, 119, 147, 157, 170, 179, 185–192], facilitates continuing medical education [119, 128, 173, 174, 193–195], inculcates moral, ethical, professional and social standards and expectations [118, 156, 160], improves patient care [29, 120, 129, 131, 135, 142, 194, 196–199] and nurtures PIF [150, 157, 172, 191, 200].

**Table 5:** Benefits of RW programs

Benefits of RW programs	Elaboration
Reflective writing supporting professional formation of physicians	Physical act of writing ° Daily writing of experiences enhanced observation skills and allowed for review of actions [157, 168, 201, 202] Improvement of self through the sharing of reflections and receiving of feedback [149, 172, 198] ° Personalised feedback for personal growth and sense of self [150, 157, 172, 200] ° Clarification of values through feedback [200, 203] Identity formation through exploration of emotions ° Acknowledgment of personal feelings and impact on clinical decisions [156, 198, 199] ° Development of empathy by reflecting upon own emotions and identifying with patients [154, 172, 204, 205] ° Acknowledgement of own coping mechanisms and vulnerability [154, 160, 206] ° Expression of humanity [156] ° Identification of morals and values, both personal and the patient’s [118, 156, 160] Identity formation through sharing of stories and experiences [137] Improving communication [115, 118, 173] ° Development of ability to relate and hence communicate with others [114] Changes in perspectives, expectations and pre-conceived assumptions [148, 149, 156, 207, 208] Areas for improvement in RW to further professional identity formation ° Reflection framework needed to most effectively improve professional decision-making [37, 191]
RW as a tool for learning enhancement	Becoming active and independent learners [96, 179, 209, 210] ° Understanding the meaning and importance of what they are learning [112, 170, 198, 207] ° Initiation of learning by consolidating past experiences and applying to future practice [174, 211] ° Asking for feedback from mentors [119, 179] ° Facilitates lifelong learning [119, 128, 173, 174, 193–195] Sharing of reflections ° Understanding other perspectives and ideas [118, 149, 153] RW as another avenue for users to engage in learning in addition to more traditional methods in classrooms ° RW assignments lend flexibility to a traditional classroom [119, 212] Integration of existing knowledge with new learning [37, 174, 197] ° By observing and reflecting on experiences to make sense of lived experiences [127, 161, 166, 174, 181, 213] ° Consolidation of learning and making connections between concepts [214, 215] Reaping the rewards of RW for learning enhancement ° Lack of appreciation for the benefits of RW for those who only completed assignments out of obligation [214] ° Too time-consuming to reflect on daily performance [119] ° Difficulty in assessing true learning potential of RW assignments, little evidence in relationship between academic achievement and reflective capacity [144, 184, 207]
RW in aiding self-understanding	Documentation of change and growth [154, 193] Increasing self-awareness [114, 127, 137, 161, 166, 179, 185, 216] ° Greater understanding of their professional role and competencies needed to fulfil responsibilities [131, 150, 174, 205, 217] ° Insights into own strengths, weaknesses and learning needs [112, 119, 150, 152, 170, 218, 219] ° Increased awareness of their own mental health with acknowledgement of fears and vulnerabilities made possible in a safe space [120, 181] ° Questioning of personal beliefs and actions [141, 153, 212, 217, 219–221] ° Making meaning in their lives [129, 166] Acknowledgement and embracing of personal emotions [166] ° Expression and confrontation of emotions they had grappled with and felt they were denied of [114, 129, 156, 172, 200, 208, 209] ° Sense of vulnerability in expression of self [160] ° Recognition of previous sense of emotional detachment [115, 129, 158] ° Emotional stability [200] Stumbling blocks for improving self-awareness ° Unfamiliarity with RW assignments increased discomfort especially with lack of support [37, 157] ° Assessments made users feel inhibited from being genuine with regards to complex situations and feelings [222]
RW enhances self-assessment	Identification of strengths and weaknesses [114, 146, 161, 193, 217] ° Promotes culture of self-monitoring and self-improvement [130, 172, 173, 185, 193, 198] ° Developing critical perspectives of self [193, 223] ° Greater ease with receiving critical feedback from others [198]
RW assists with development of clinical behaviour and patient care	Improved communication skills between healthcare professionals and with patients [29, 131, 142, 194, 196–198] ° Realised importance of interprofessional teamwork [131, 135, 197] ° Improved skill in breaking bad news [129, 199] ° Improved skill in active listening [120] Improved clinical reasoning and decision making [118, 126, 194, 196, 199, 200] ° Reflection on clinical situations or incidents to rationalise behaviour retrospectively [37, 174, 177, 224, 225] ° Reflection in action [226] Development of soft skills ° Development of empathy [38, 127, 158, 185, 197, 200, 205, 219, 227] Patient-centred care [131, 212] ° To be more aware of patient autonomy and respecting each individual’s wishes [118, 129, 131, 228] ° Realised importance of trust in doctor-patient relationship [171, 198, 205] ° Improvement in patient outcomes [195]

From a personal perspective, RW increases self-awareness [114, 127, 137, 161, 166, 179, 185, 202, 216], self-advancement [9, 131, 134, 150, 168, 174, 195, 205, 217, 229], facilitates understanding of individual strengths, weaknesses and learning needs [112, 119, 150, 152, 170, 218, 219], promotes a culture of self-monitoring, self-improvement [130, 172, 173, 185, 193, 198, 201, 210, 211], developing critical perspectives of self [193, 223] and nurtures resilience and better coping [154, 160, 206]. RW also guides shifts in thinking and perspectives [148, 149, 156, 203, 207, 208] and focuses on a more holistic appreciation of decision-making [37, 118, 126, 174, 177, 194, 196, 199, 200, 224–226] and their ramifications [37, 112, 116, 130, 131, 141, 154, 179, 193, 194, 196, 204, 207, 218, 230].

Table 6 combines current lists of the shortcomings of RW. These limitations may be characterised by individual, structural and assessment styles.

**Table 6:** Shortcomings of RW programs

Shortcomings of RW programs	Elaboration
Problems found in implementation of RW curriculum	Anxiety with having their private thoughts being shared with others ° Preference for one-on-one sharing with tutors instead [129, 149, 209, 231] ° Censorship of thoughts and reflections when sharing with others [37, 114, 136, 149, 160, 183] ° Process of sharing could feel impersonal if sharing is done virtually [165] May fail to cater to the different learning styles of users [220, 232] ° Query as to the extent that writing may be able to capture elements of the users’ reflective processes [118] ° Other modalities for reflection (e.g. blogging) might have greater appeal to users [120] ° RW too restrictive for more experienced users due to rigidity of suggested frameworks [142, 196] Barriers to user participation ° Lack of time and fatigue [29, 119, 136, 138, 157, 161, 167–169, 176, 181, 193, 196, 226, 232, 233] ° Lack of self-direction and motivation [29, 79, 119, 176, 188, 226, 231] ° Difficulty dealing with negative emotions arising from reflecting on difficult events [114, 168, 176, 193, 230] ° Felt that RW was unnecessary as they were already adept at introspection [227] Objectives were not clearly defined to users and assessors ° Greater clarity of goals of RW needed throughout course for users to understand importance of what they were doing [114, 129, 135, 138, 142, 209, 227] ° Greater emphasis to be placed on role of assessors for them to provide adequate feedback and mentorship for users [50, 138]
Factors affecting quality of reflection	Lack of confidentiality and trust resulting in censorship of genuine thoughts and reflections [37, 114, 136, 149, 169, 183, 196] Lack of support and feedback from mentors [37, 119, 133, 196] Problems relating to writing ° Language competencies affecting expression [167, 229] ° Learning to write in a new voice unlike academic writing [114, 136] Decreased authenticity of reflections to meet expectations of graded curriculum [9, 115, 157, 161, 166, 193, 209, 234] Did not take module seriously due to it being formatively assessed [114, 172, 182, 226] Enforcing of daily reflections caused users to reflect on experiences that were insignificant [119, 235, 236]
Problems found with assessment of RW curriculum	Assessment distracts users from the essence of reflection ° Grading pressures users to write for approval [114, 115, 118, 129, 138, 143, 149, 155, 157, 209, 232, 237, 238] ° Assessment causes censorship of tension of ethical dilemmas or censorship of unconventional opinions [119, 209] Faculty’s confusion with assessment of reflection ° Uncomfortable with idea of reflection due to lack of experience [115, 226] ° Inconsistent definitions of reflections [114, 133, 188, 237] ° Subjective nature of judging the content [237] ° Influence of writing ability [132, 174, 180, 183] ° Lack of confidence in correlating assessment grade with depth of reflection [29, 105, 118, 126, 177, 207] Problems with rubrics ° Unclear rubric categories with overlaps between different levels [145] ° Difficulty maintaining a consistent high inter-rater variability [143, 239]
Possible problems with reflection in itself	Triggering of negative emotions which users are unable to escape ° Questioning what has always been instinctual knowledge or status quo might bring instead a sense of uncertainty which complicates decision-making [207, 240] ° Users might become overly critical of themselves [207, 241] ° Self-doubt [225] Becoming negatively self-isolated ° Personal forms of critical reflection might have the unintended effect of users becoming too focused on themselves instead [207] Could distract learners from spending time on technical skills or knowledge acquisition [207, 225]

It is suggested that RW does not cater to the different learning styles [220, 232], cultures [190], roles, values, processes and expectations of RW [114, 129, 135, 138, 142, 209, 227, 234], and physicians' differing levels of self-awareness [29, 79, 119, 176, 188, 226, 231, 236], motivations [29, 119, 136, 138, 157, 161, 167–169, 176, 181, 193, 196, 226, 232, 233] and willingness to engage in RW [37, 114, 136, 149, 160, 183]. RW is also limited by poorly prepared physicians and misaligned expectations whilst a lack of privacy and a safe setting may precipitate physician anxiety at having their private thoughts shared [129, 149, 209, 231]. RW is also compromised by a lack of faculty training [143, 145, 239], mentoring support [37, 50, 119, 133, 196] and personalised feedback [50, 114, 136, 167, 229] which may lead to self-censorship [37, 114, 136, 149, 160, 183] and an unwillingness to address negative emotions arising from reflecting on difficult events [114, 168, 176, 193, 230], circumventing the reflective process [118, 142, 165, 196] .

Variations in assessment styles [9, 115, 157, 161, 166, 193, 209], depth [29, 105, 118, 126, 177, 207] and content [37, 114, 136, 149, 169, 183, 196], and pressures to comply with graded assessments [114, 115, 118, 129, 138, 143, 149, 155, 157, 209, 232, 237, 238] also undermine efforts of RW.

### Domain 4. Recommendations

In the face of practice variations and challenges, there have been several recommendations on improving practice.

#### Boosting awareness of RW

Acknowledging the importance of a physician’s motivations, willingness and judgement [37], an RW program must acquaint physicians with information on RW’s role [128], program expectations, the form, frequency and assessments of RW and the support available to them [130, 132, 150, 154, 242] and its benefits to their professional and personal development [96, 227] early in their training programs [115, 220, 242, 243]. Physicians should also be trained on the knowledge and skills required to meet these expectations [1, 37, 135, 151, 160, 215, 244, 245].

#### A structured program and environment

Recognising that effective RW requires a structured program. Recommendations focus on three aspects of the program design [132]. One is the need for trained faculty [9, 115, 219, 220, 230, 233, 242, 246], accessible communications, protected time for RW and debriefs [125], consistent mentoring support [190] and assessment processes [247]. This will facilitate trusting relationships between physicians and faculty [30, 114, 168, 196, 231, 233]. Two, the need to nurture an open and trusting environment where physicians will be comfortable with sharing their reflections [96, 128], discussing their emotions, plans [127, 248] and receiving feedback [9, 37, 79, 114, 119, 128, 135, 173, 176, 179, 190, 237]. This may be possible in a decentralised classroom setting [163, 190]. Three, RW should be part of the formal curriculum and afforded designated time. RW should be initiated early and longitudinally along the training trajectory [116, 122].

#### Adjuncts to RW programs

Several approaches have been suggested to support RW programs. These include collaborative reflection, in-person discussion groups to share written reflections [128, 131, 138, 196, 199, 231, 249] and reflective dialogue to exchange feedback [119], use of social media [149, 160, 169, 194, 204, 230], video-recorded observations and interactions for users to review and reflect on later [133]. Others include autobiographical reflective avenues in addition to practice-oriented reflection [137], support groups to help meditate stress or emotions triggered by reflections [249] and mixing of reflective approaches to meet different learning styles [169, 250].

## Discussion

In answering the primary research question, *“How is reflective writing structured, assessed and supported in medical education?”*, this SSR in SEBA highlights several key insights. To begin, RW involves integrating the insights of an experience or point of reflection (henceforth ‘event’) into the physician’s currently held values, beliefs and principles (henceforth belief system). Recognising that an ‘event’ has occurred and that it needs deeper consideration highlights the physician’s *sensitivity*. Recognising the presence of an ‘event’ triggers an evaluation as to the urgency in which it needs to be addressed, where it stands amongst other ‘events’ to be addressed and whether the physician has the appropriate skills, support and time to address the ‘event’. This reflects the physician’s *judgement*. The physician must then determine whether they are *willing* to proceed and the ramifications involved. These include ethical, medical, clinical, administrative, organisational, sociocultural, legal and professional considerations. This is then followed by contextualising them to their own personal, psychosocial, clinical, professional, research, academic, and situational setting. Weighing these amidst competing ‘events’ underlines the import of the physician’s ability to *‘balance’* considerations. Creating and experimenting on their ‘working hypothesis’ highlights their *‘ability’,* whilst how they evaluate the effects of their experimentation and how they adapt their practice underscores their ‘*responsiveness*’ [2, 5, 74, 75, 77, 78, 85–87, 90].

The concepts of ‘*sensitivity’, ‘judgement’, ‘willingness’, ‘balance’, ‘ability’ and ‘responsiveness’* spotlight environmental and physician-related factors. These include the physician’s motivations, knowledge, skills, attitudes, competencies, working style, needs, availabilities, timelines, and their various medical, clinical, administrative, organisational, sociocultural, legal, professional, personal, psychosocial, clinical, research, academic and situational experiences. It also underlines the role played by the physician’s beliefs, moral values, ethical principles, familial mores, cultural norms, attitudes, thoughts, decisional preferences, roles and responsibilities. The environmental-related factors include the influence of the curriculum, the culture, structure, format, assessment and feedback of the RW process and the program it is situated in. Together, the physician and their environmental factors not only frame RW as a sociocultural construct necessitating holistic review but also underscore the need for longitudinal examination of its effects. This need for holistic and longitudinal appraisal of RW is foregrounded by the experimentations surrounding the ‘working hypothesis’ [2, 5, 72, 74, 77, 84–86, 90]. In turn, experimentations and their effects affirm the notion of regular use of RW and reiterate the need for longitudinal reflective relationships that provide guidance, mentoring and feedback [87, 90]. These considerations set the stage for the proffering of a new conceptual model of RW.

**Fig. 3:**
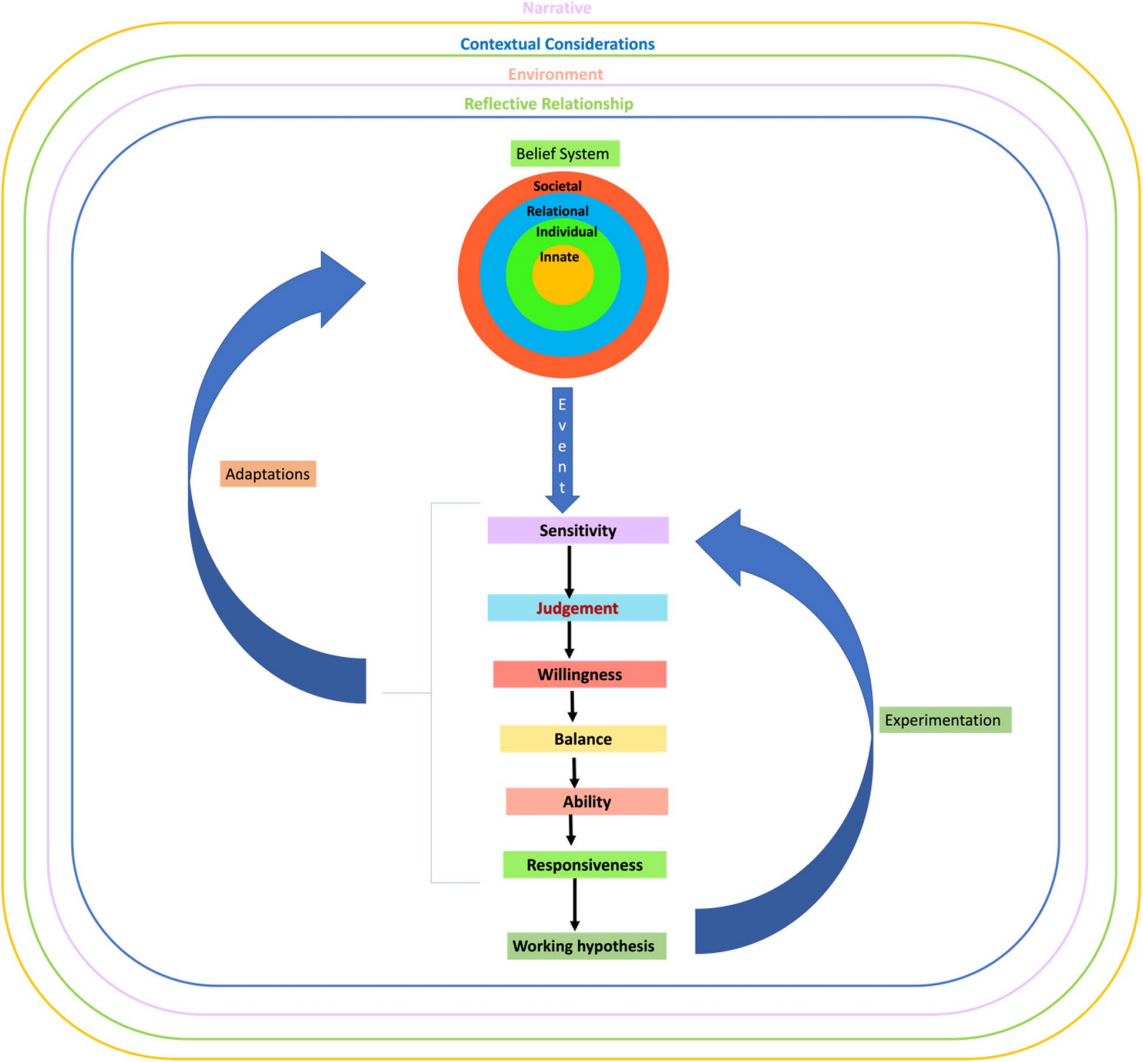
Krishna Model of Reflective Writing

To begin, the Krishna Model of Reflective Writing (Fig. 3) builds on the Krishna-Pisupati Model [10] used to describe evaluations of professional identity formation (PIF) [8, 10, 24, 251]. Evidenced in studies of how physicians cope with death and dying patients, moral distress and dignity-centered care [46, 54], the Krishna-Pisupati Model suggests that the physician’s belief system is informed by their self-concepts of personhood and identity. This is effectively characterised by the Ring Theory of Personhood (RToP) [11].

The Krishna Model of RW posits that the RToP is able to encapsulate various aspects of the physician’s belief system. The Innate Ring which represents the innermost ring of the four concentric rings depicting the RToP is derived from currently held spiritual, religious, theist, moral and ethical values, beliefs and principles [13, 51, 53, 252]. Encapsulating the Innate Ring is the Individual Ring. The Individual Ring’s belief system is derived from the physician’s thoughts, conduct, biases, narratives, personality, decision-making processes and other facets of conscious function which together inform the physician’s Individual Identity [13, 51, 53, 252]. The Relational Ring is shaped by the values, beliefs and principles governing the physician’s personal and important relationships [13, 51, 53, 252]. The Societal Ring, the outermost ring of the RToP is shaped by regnant societal, religious, professional and legal expectations, values, beliefs and principles which inform their interactions with colleagues and acquaintances [13, 51, 53, 252]. Adoption of the RToP to depict this belief system not only acknowledges the varied aspects and influences that shape the physician’s identity but that the belief system evolves as the physician’s environment, narrative, context and relationships change.

The environmental factors influencing the belief system include the support structures used to facilitate reflections such as appropriate protected time, a consistent format for RW, a structured assessment program, a safe environment, longitudinal support, timely feedback and trained faculty. The Krishna Model of RW also recognises the importance of the relationships which advocate for the physician and proffer the physician with coaching, role modelling, supervision, networking opportunities, teaching, tutoring, career advice, sponsorship and feedback upon the RW process. Of particular importance is the relationship between physician and faculty (henceforth reflective relationship). The reflective relationship facilitates the provision of personalised, appropriate, holistic, and frank communications and support. This allows the reflective relationship to support the physician as they deploy and experiment with their ‘working hypothesis’. As a result, the Krishna Model of RW focuses on the dyadic reflective relationship and acknowledges that there are wider influences beyond this dyad that shape the RW process. This includes the wider curriculum, clinical, organisational, social, professional and legal considerations within specific practice settings and other faculty and program-related factors. Important to note, is that when an ‘event’ triggers ‘*sensitivity’, ‘judgement’, ‘willingness’, ‘balance’, ‘ability’ and ‘responsiveness’, *the process of creating and experimenting with a ‘working hypothesis' and adapting one's belief system is also shaped by the physician’s narratives, context, environment and relationships. 

In answering its secondary question, “*How might a reflective writing program in medical education be structured?*”, the data suggests that an RW program ought to be designed with due focus on the various factors influencing the physician's belief system, their *‘sensitivity’, ‘judgement’, ‘willingness’, ‘balance’, ‘ability’ and ‘responsiveness’, and their* creation and experimentation with their ‘working hypothesis’. These will be termed the *‘physician's reactions’*. The design of the RW program ought to consider the following factors:

- Belief system
  i. Narratives
    1. Recognising that the physician’s notion of ‘*sensitivity’, ‘judgement’, ‘willingness’, ‘balance’, ‘ability’ and ‘responsiveness’* is influenced by their experience, skills, knowledge, attitude and motivations, physicians recruited to the RW program should be carefully evaluated
    2. To align expectations, the physician should be introduced to the benefits and role of RW in their personal and professional development
    3. The ethos, frequency, goals and format of the reflection and assessment methods should be clearly articulated to the physician [253]
    4. The physician should be provided with the knowledge, skills and mentoring support necessary to meet expectations [76, 79, 105, 123, 124, 254, 255]
    5. Training and support must also be personalised
  ii. Contextual considerations
    1. Recognising that the physician’s academic, personal, research, administrative, clinical, professional, sociocultural and practice context will change, the structure, approach, assessment and support provided must be flexible and responsive
    2. The communications platform should be easily accessible and robust to attend to the individual needs of the physician in a timely and appropriate manner
    3. The program must support diversity [207]
  iii. Environment
    1. The reflective relationship is shaped by the culture and structure of the environment in which the program is hosted in
    2. The RW programs must be hosted within a formal structured curriculum, supported and overseen by a host organisation which is able to integrate the program into regnant educational and assessment processes [9, 115, 219, 220, 230, 233, 242, 246]
  iv. Reflective relationship
    1. The faculty must be trained and provided access to counselling, mindfulness meditation and stress management programs [249]
    2. The faculty must support the development of the physician’s metacognitive skills [256–259], and should create a platform that facilitates community-centered learning [173, 176], structured, timely, personalised open feedback [119, 135, 179, 237] and support [128, 131, 138, 196, 199, 231, 249]
    3. The faculty must be responsive to changes and provide appropriate personal, educational and professional support and adaptations to the assessment process when required [207]
    4. To facilitate the development of effective reflective relationships, a consistent faculty member should work with the physician and build a longitudinal trusting, open and supportive reflective relationship
- Physician’s reactions
  1. The evolving nature of the various structures and influences upon the RW process underscores the need for longitudinal assessment and support
  2. The physician must be provided with timely, appropriate and personalised training and feedback
  3. The program’s structure and oversight must also be flexible and responsive
  4. There must be accessible longitudinal mentoring support
  5. The format and assessment of RW must account for growing experience and competencies as well as changing motivations and priorities
  6. Whilst social media may be employed to widen sharing [149, 155, 160, 169, 194], privacy must be maintained [120, 189]

### On assessment

1. Assessment rubrics should be used to guide the training of faculty, education of physicians and guidance of reflections [37, 116–122]
2. Assessments ought to take a longitudinal perspective to track the physician's progress [116, 122]

Based on the results from this SSR in SEBA, we forward a guide catering to novice reflective practitioners (Additional file 1).

### Limitations

This SSR in SEBA suggests that, amidst the dearth of rigorous quantitative and qualitative studies in RW and in the presence of diverse practices, approaches and settings, conclusions may not be easily drawn. Extrapolations of findings are also hindered by evidence that appraisals of RW remain largely reliant upon single time point self-reported outcomes and satisfaction surveys.

## Conclusion

This SSR in SEBA highlights a new model for RW that requires clinical validation. However, whilst still not clinically proven, the model sketches a picture of RW’s role in PIF and the impact of reflective processes on PIF demands further study. As we look forward to engaging in this area of study, we believe further research into the longer-term effects of RW and its potential place in portfolios to guide and assess the development of physicians must be forthcoming.

## Supplementary Information

**Additional file 1.** Guide to Reflective Writing.

## Data Availability

All data generated or analysed during this review are included in this published article and its supplementary files.

## Abbreviations

RW: Reflective Writing
PIF: Professional Identity Formation
RToP: Ring Theory of Personhood
BEME: Best Evidence Medical Education
STORIES: Structured approach to the Reporting In healthcare education of Evidence Synthesis
PRISMA: Preferred Reporting Items for Systematic Reviews and Meta-Analyses
SSR: Systematic Scoping Review
SEBA: Systematic Evidence-Based Approach
YLLSoM: Yong Loo Lin School of Medicine
PICOs: Population, Intervention, Comparison, Outcome, Study Design
RAMESES: Realist And Meta-narrative Evidence Syntheses - Evolving Standards
