# Supplementary material for: A systematic scoping review of reflective writing in medical education
Source: BMC Med Educ. 2023 Jan 9;23:12. doi: 10.1186/s12909-022-03924-4 (PMC9830881; doi:10.1186/s12909-022-03924-4)
Supplement: Supplementary file 1 — Additional file 1. Guide to Reflective Writing. [file 12909_2022_3924_MOESM1_ESM.docx]

**Additional file 1. Guide to Reflective Writing**

**Guide to Effective Reflective Writing for Medical Students**

*This guide caters to novice reflective practitioners. The aim of this guide is to provide (i) basic understanding of reflection and reflective writing, grounded in theory, (ii) purpose of reflective writing for medical practitioners and (iii) instructions on how one reflective cycle should be conducted and how various reflective models can assist in reflective thinking and writing.*

1. Reflective writing helps:
   - 1. Professional identity formation (Mann et al., 2009) through exploration of personal emotions, morals and values (Ament Giuliani Franco et al., 2020; Eutsler & Curcio, 2019; Vicini et al., 2017)
     2. As a means of enhancing learning, metacognition is needed for metamemory, metacomprehension, problem-solving, and critical thinking (Mann et al., 2009) through the integration of existing knowledge with new learning (Karnieli-Miller et al., 2018; Murdoch-Eaton & Sandars, 2014; Olex et al., 2020) and making sense of lived experiences (Eriksen, 2012; Freeman, 2001; Herrick-Reynolds et al., 2019; Janssen et al., 2009; Karnieli-Miller et al., 2018; Pavlovich et al., 2009)
     3. Self-understanding (Mann et al., 2009) through increased awareness of individual strengths, weaknesses and learning needs (Chuang et al., 2013; Embo et al., 2014; Grant et al., 2006; Kanthan & Senger, 2011; Liu et al., 2016; Standal & Rugseth, 2014; Vettraino et al., 2019) and through the provision of a safe place to acknowledge fears and vulnerabilities (Herrick-Reynolds et al., 2019; Kasman, 2004)
     4. Improving patient care (Mann et al., 2009) through improved communication skills (Adams et al., 2006; Bjerkvik & Hilli, 2019; Chretien et al., 2012; Nagano et al., 2019; Olex et al., 2020; Tsingos et al., 2014; Winkel et al., 2017), clinical reasoning and decision-making skills (Ament Giuliani Franco et al., 2020; Bjerkvik & Hilli, 2019; Carr & Carmody, 2006; Chretien et al., 2012; Shapiro et al., 2006; Tsingos et al., 2015).
     5. Points (i) to (iv) are eventual and desired outcomes of reflection, which are hard to measure. More proximal are the skills that we hope to develop through reflective writing – reflective capacity, competence, reflective practice (habit).
2. Before starting reflective writing/ reflection, one should have the correct mindset
3. The proposed template is as such (Dalsgaard, 2020; Eutsler & Curcio, 2019; Goldman & Grimbeek, 2015; Goodyear et al., 2013; Murdoch-Eaton & Sandars, 2014; Naeger et al., 2015; Tawanwongsri & Phenwan, 2019; Wang & Liao, 2020).
   1. Description of events (the ‘what’) this may be a memorable feeling, thought or event, which can be positive or negative. It includes description of behavior, ideas, and feelings during the experience (What did you feel? How did you behave? What were your thoughts during the event?) [There is a need to emphasize that this description is factual, dispassionate, and taken from a 3^rd^ person perspective.]
   2. PAUSE AND STEP BACK- Identify what about the situation that made you feel uncertain?
   3. Deconstructing event (the ‘so what’)
      1. Firstly, the making sense or “so why”.
         1. Studying conditions of situation and reasoning and rationalizing behaviour, ideas, emotions and assumptions made during the event. (Why did one act the way one did? Why did one feel the way one did?)
      2. Secondly, the making meaning or “so what”.
         1. What does this mean to me as a person?
         2. What does this mean to me professionally?
         3. What does this mean to the immediate others (patient, family, co-workers)?
      3. Relating current experience to other similar experiences in the past to find connections (Any past experiences similar to current event? What were the similarities/ differences?)
      4. Re-examining experience from other’s perspectives (What were the consequences of your behaviour? How did the other person feel?)
   4. Learning outcomes (After making an analysis (sense & meaning), now we evaluate what we have learnt.)
      1. Learning from experience and changed perspectives and actions (What was learned? Why is this important? How have I made sense of this experience?)
      2. Future action plan (If this arose again, what would you do?)
   5. Extra: Framing (Now we apply what we have learnt: reframing of perspectives, future actions, other situations, the broader society and world - transformation.)
      1. explore the role of gender, religion, age, education, and spirituality in explaining or learning from their experiences.
4. Students should consider sharing reflections with their peers or a trusted mentor to obtain feedback (Xiao et al., 2016). Feedback in general helps to challenge assumptions made unknowingly by students and to point out new perspectives and considerations for students (Murdoch-Eaton & Sandars, 2014). (Here the term facilitation rather than feedback is used. Feedback is essentially telling the learner where the gap is and what to do about it. Facilitation is to help the learner self-identify the gap and develop the skills to address the gap. Successful facilitation requires – trust & safety, skilled faculty (asking provoking questions, inquiring questions, perspective-challenging questions), curious but non-judgmental attitude, confidentiality, transparency, etc.)

**Guide to Assessing Reflective Writing for Tutors**

*This guide caters to tutors who are new to the reflective writing curriculum. The aim of this guide is to provide (i) Understanding of the various models assessing reflective writing by breaking them down into 3 distinct categories, (ii) Explanation of the important mindset to have when assessing reflective writing, and (iii) Possible adjuncts to be in place in a reflective writing curriculum to provide additional support for teachers.*

1. Teachers would first need to understand the meaning of reflection, process of reflection and adopt reflection skills before becoming assessors and facilitators for reflective writing programmes (Zoellner et al., 2017)
   1. Teachers would require adequate training in terms of training of own self-reflection skills (Collin et al., 2013; Ekebergh, 2007; Sukhato et al., 2016) to allow supervisors to be comfortable with the idea of reflection with own personal experience of reflective writing (Hudson et al., 2012; Sukhato et al., 2016; Too, 2013)
   2. Definitions of reflections would have to be consistent for teachers to confidently assess reflection (Hayton et al., 2015; Jamil & Hamre, 2018; Nguyen et al., 2014; Pavlovich, 2007)
2. Reflective writing can be categorized into the following for assessment (Kember, 1999; Mezirow, 1990; Thorpe, 2004; Wong, 1997):
   1. Non-reflectors (i.e. Habitual reflection, thoughtful action, introspection)
      1. Habitual reflection: Only refers to what was previously learned and now continues to use it automatically through constant use
      2. Thoughtful action: attempts to apply pre-existing knowledge without any attempt to re-assess appropriateness. Assumptions and pre-existing thoughts and values remain unchanged
      3. Introspection: Description of thoughts and feelings but no attempt to re-examine the validity or rationalize feelings
      4. **Non-reflectors**: Writing which only contains pure description of thoughts, feelings and values, with no reassessment of assumptions made. No rationalization of actions and behaviour is made.
   2. Reflectors (i.e. content reflection, process reflection, content and process reflection)
      1. Content reflection: Rationalizing thoughts, feelings, actions, and behaviour during the event
      2. Process reflection: Rationalizing how one has performed
      3. **Reflectors**: Analysis of thoughts, feeling and actions during the event. Involvement of meaning making and examination of experience.
   3. Critical reflectors (i.e. Premise reflection) ) (Those who can make sense, make meaning, evaluate are “reflectors’. But those who can apply, transform, and extend what they learnt to other situations and the broader society are “critical reflectors”.)
      1. Premise reflection: Involves putting aside of pre-existing assumptions and making of action plan for future events
      2. **Critical reflectors**: involvement of a change in perspectives to be applied in future events
   4. Tutor must be clear with the distinction between the three levels of reflection. If transformed into rubric categories, overlaps between the different levels can cause confusion in assessment (Miller-Kuhlmann et al., 2016).
   5. Performance on one reflective writing sample is not strongly indicative of the overall reflective writing skill of the student. When using the REFLECT tool for example, approximately 14 writing samples were required to achieve reasonable inter-sample reliability to draw meaningful conclusions about the student’s reflective capacity. (Moniz et al., 2015).
3. Things to note for teachers when grading reflective writing:
   1. Grading pressures can distract users from the essence of reflection (Ament Giuliani Franco et al., 2020; Brown et al., 2020; Bruno & Dell'Aversana, 2017; Cox, 2005; Hayton et al., 2015; Martin, 2005; Moniz et al., 2015; Pavlovich, 2007; Rees et al., 2005; Roberts, 2016; Rosenbaum et al., 2005; Shaughnessy et al., 2017; Sukhato et al., 2016). Tutors should keep in mind that the focus of reflective writing programs is to promote the culture of reflection in students and writing is the modality through which students can achieve critical reflection, and not simply a measure through which students are assessed on their reflective skills (Charon & Hermann, 2012). Assessment should be a way of tracking development of students’ reflective writing skills and providing structure and foundation for students in development of these skills, rather than for simple grading.
   2. Tutors must be aware that reflective writing cannot be reduced to checkboxes or summative evaluation of what students have or have not accomplished. Focus should instead be on meaning making, with tutors facilitating the progress of students towards critical reflection (Wear et al., 2012). Tutors must understand that reflective writing assessment is different from the technical assessment marking scheme which they are more used to (Pavlovich, 2007).
   3. Tutors could modify rubrics and weightages of assessment components based on expected competency and experience of student being (Ryan & Ryan, 2013; Sweet et al., 2019). For example, in longitudinal reflective writing programs, different weightages could be applied to final year students to assess a higher level of reflective writing expected for them, with increased focus on achieving critical thinking.
4. Feedback should be synonymous with assessment in reflective writing programs.
   1. Tutors would require training to acquire the correct facilitation skills to probe deeper levels of reflection to aid students in considering different perspectives (Glod et al., 2016; Standal & Rugseth, 2014).
   2. Feedback given should include reflection-inviting questions and the use of ‘coaching rather than evaluative language’(Wald et al., 2010).
   3. Fostering of mentor-mentee relationship is crucial and key to reflective writing programs in order to build trust between mentor and mentees. Tutors should
      1. Maintain confidentiality to allow students to be honest and open in their reflections (Thorpe, 2004)
      2. Or could consider sharing their own reflections with their mentees (Henderson et al., 2002)
5. A support system for teachers could be set up for training of staff members
   1. Support system for training of self-reflection skills for tutors themselves (Collin et al., 2013; Ekebergh, 2007; Sukhato et al., 2016).
   2. ‘Super mentor’ system where faculty members skilled in reflective writing can review and give feedback to other faculty members involved (Arntfield et al., 2016; Sukhato et al., 2016).

**References**

Adams, C. L., Nestel, D., & Wolf, P. (2006). Reflection: a critical proficiency essential to the effective development of a high competence in communication. *J Vet Med Educ*, *33*(1), 58-64. <https://doi.org/10.3138/jvme.33.1.58>

Ament Giuliani Franco, C., Franco, R. S., Cecilio-Fernandes, D., Severo, M., Ferreira, M. A., & de Carvalho-Filho, M. A. (2020). Added value of assessing medical students' reflective writings in communication skills training: a longitudinal study in four academic centres. *BMJ Open*, *10*(11), e038898. <https://doi.org/10.1136/bmjopen-2020-038898>

Arntfield, S., Parlett, B., Meston, C. N., Apramian, T., & Lingard, L. (2016). A model of engagement in reflective writing-based portfolios: Interactions between points of vulnerability and acts of adaptability. *Med Teach*, *38*(2), 196-205. <https://doi.org/10.3109/0142159x.2015.1009426>

Bjerkvik, L. K., & Hilli, Y. (2019). Reflective writing in undergraduate clinical nursing education: A literature review. *Nurse Educ Pract*, *35*, 32-41. <https://doi.org/10.1016/j.nepr.2018.11.013>

Brown, A., Jauregui, J., Ilgen, J. S., Riddell, J., Schaad, D., Strote, J., & Shandro, J. (2020). Does the Medium Matter? Evaluating the Depth of Reflective Writing by Medical Students on Social Media Compared to the Traditional Private Essay Using the REFLECT Rubric. *West J Emerg Med*, *21*(1), 18-25. <https://doi.org/10.5811/westjem.2019.11.44263>

Bruno, A., & Dell'Aversana, G. (2017). Reflective Practice for Psychology Students: The Use of Reflective Journal Feedback in Higher Education. *Psychology Learning and Teaching*, *16*(2), 248-260. <http://libproxy1.nus.edu.sg/login?url=https://search.proquest.com/docview/1969021725?accountid=13876&bdid=6923&_bd=jArmK7vFBbDKLGVU%2FXGCa1x3IIY%3D>

Carr, S., & Carmody, D. (2006). Experiential learning in women's health: medical student reflections. *Med Educ*, *40*(8), 768-774. <https://doi.org/10.1111/j.1365-2929.2006.02536.x>

Charon, R., & Hermann, N. (2012). Commentary: a sense of story, or why teach reflective writing? *Acad Med*, *87*(1), 5-7. <https://doi.org/10.1097/ACM.0b013e31823a59c7>

Chretien, K. C., Chheda, S. G., Torre, D., & Papp, K. K. (2012). Reflective writing in the internal medicine clerkship: a national survey of clerkship directors in internal medicine. *Teach Learn Med*, *24*(1), 42-48. <https://doi.org/10.1080/10401334.2012.641486>

Chuang, L. L., Liu, H. W., Lin, Y. C., Wang, Y. W., & Chu, S. Y. (2013). Using structured narrative to help a medical student reflect on an unexpected clinical situation [Article]. *Tzu Chi Medical Journal*, *25*(1), 68-70. <https://doi.org/10.1016/j.tcmj.2012.05.011>

Collin, S., Karsenti, T., & Komis, V. (2013). Reflective practice in initial teacher training: Critiques and perspectives. *Reflective Practice*, *14*(1), 104-117.

Cox, E. (2005). Adult learners learning from experience: Using a reflective practice model to support work-based learning. . *Reflective Practice*, *6*(4), 459-472.

Dalsgaard, C. (2020). Reflective Mediation: Toward a Sociocultural Conception of Situated Reflection. *Frontline Learning Research*, *8*(1), 1-13. <http://libproxy1.nus.edu.sg/login?url=https://search.proquest.com/docview/2396851366?accountid=13876&bdid=6923&_bd=zMtVw6Ebi94DSqJbMVJ5Jo8cNCo%3D>

Ekebergh, M. (2007). Lifeworld-based reflection and learning: A contribution to the reflective practice in nursing and nursing education. . *Reflective Practice*, *8*(3), 331-343.

Embo, M. P., Driessen, E., Valcke, M., & Van Der Vleuten, C. P. (2014). Scaffolding reflective learning in clinical practice: a comparison of two types of reflective activities. *Med Teach*, *36*(7), 602-607. <https://doi.org/10.3109/0142159x.2014.899686>

Eriksen, M. (2012). Facilitating Authentic Becoming. *Journal of Management Education*, *36*(5), 698-736. <http://libproxy1.nus.edu.sg/login?url=https://search.proquest.com/docview/1140138744?accountid=13876&bdid=6923&_bd=SLOAhHgILSUPs2tvV8jcFuWG55A%3D>

Eutsler, L., & Curcio, R. (2019). Private blog reflections connecting course content with field experiences: Preservice teachers grapple with teacher identity., *20*(2), 250-265.

Freeman, M. (2001). Reflective logs: an aid to clinical teaching and learning. *Int J Lang Commun Disord*, *36 Suppl*, 411-416. <https://doi.org/10.3109/13682820109177921>

Glod, S. A., Richard, D., Gordon, P., Fecile, M. L., Kees-Folts, D., Kreher, M., Moser, E. M., Wolpaw, D. R., Yang, C., & Haidet, P. (2016). A Curriculum for Clerkship Students to Foster Professionalism Through Reflective Practice and Identity Formation. *MedEdPORTAL*, *12*, 10416. <https://doi.org/10.15766/mep_2374-8265.10416>

Goldman, J. D. G., & Grimbeek, P. (2015). Pre-service primary school teachers' self-reflective competencies in their own teaching. *European Journal of Psychology of Education*, *30*(2), 189-207.

Goodyear, H. M., Bindal, T., & Wall, D. (2013). How useful are structured electronic portfolio templates to encourage reflective practice? *Med Teach*, *35*(1), 71-73. <https://doi.org/10.3109/0142159x.2012.732246>

Grant, A., Kinnersley, P., Metcalf, E., Pill, R., & Houston, H. (2006). Students' views of reflective learning techniques: an efficacy study at a UK medical school. *Med Educ*, *40*(4), 379-388. <https://doi.org/10.1111/j.1365-2929.2006.02415.x>

Hayton, A., Kang, I., Wong, R., & Loo, L. K. (2015). Teaching medical students to reflect more deeply. *Teaching and Learning in Medicine*, *27*(4), 410-416.

Henderson, E., Berlin, A., Freeman, G., & Fuller, J. (2002). Twelve Tips for Promoting Significant Event Analysis To Enhance Reflection in Undergraduate Medical Students. In (Vol. 24, pp. 121-124).

Herrick-Reynolds, K., Sewanan, L. R., Zheng, D. J., Wang, P., Encandela, J., Shahu, A., & Reisman, A. (2019). A novel near-peer reflective writing workshop. *Clin Teach*, *16*(4), 339-344. <https://doi.org/10.1111/tct.13057>

Hudson, J. N., Rienits, H., Corrin, L., & Olmos, M. (2012). An innovative OSCE clinical log station: a quantitative study of its influence on Log use by medical students. *BMC Med Educ*, *12*, 111. <https://doi.org/10.1186/1472-6920-12-111>

Jamil, F. M., & Hamre, B. K. (2018). Teacher Reflection in the Context of an Online Professional Development Course: Applying Principles of Cognitive Science to Promote Teacher Learning. *Action in Teacher Education*, *40*(2), 220-236. <http://libproxy1.nus.edu.sg/login?url=https://search.proquest.com/docview/2101383899?accountid=13876&bdid=6923&_bd=VAJcS7ws6kG26N2r8pJNWv2qpuA%3D>

Janssen, F., de Hullu, E., & Tigelaar, D. (2009). Using a Domain-Specific Model to Improve Student Teachers' Reflections on Positive Teaching Experiences. *Action in Teacher Education*, *31*(2), 86-98. <http://libproxy1.nus.edu.sg/login?url=https://search.proquest.com/docview/61821726?accountid=13876&bdid=6923&_bd=gW9CVQ%2Fpy%2FdqSxsw5IyWGRn%2BkAk%3D>

Kanthan, R., & Senger, J. L. (2011). An appraisal of students' awareness of "self-reflection" in a first-year pathology course of undergraduate medical/dental education. *BMC Med Educ*, *11*, 67. <https://doi.org/10.1186/1472-6920-11-67>

Karnieli-Miller, O., Palombo, M., & Meitar, D. (2018). See, reflect, learn more: Qualitative analysis of breaking bad news reflective narratives. *Medical education*, *52*(5), 497-512.

Kasman, D. L. (2004). "Doctor, are you listening?" A writing and reflection workshop. *Fam Med*, *36*(8), 549-552.

Kember, D., Jones, A., Loke, A., McKay, J., Sinclair, K., Tse, H., Webb, C., Wong, F., Wong, M. & Yeung, E. (1999). Determining the level of reflective thinking from students’ written journals using a coding scheme based on the work of Mezirow. *International Journal of Lifelong Education*, *18*(1), 18-30.

Liu, G. Z., Jawitz, O. K., Zheng, D., Gusberg, R. J., & Kim, A. W. (2016). Reflective Writing for Medical Students on the Surgical Clerkship: Oxymoron or Antidote? *J Surg Educ*, *73*(2), 296-304. <https://www.ncbi.nlm.nih.gov/pmc/articles/PMC5536974/pdf/nihms872600.pdf>

Mann, K., Gordon, J., & MacLeod, A. (2009). Reflection and reflective practice in health professions education: A systematic review. *Adv in Health Sci Educ*, *14*(4), 595-621.

Martin, M. (2005). Reflection in Teacher Education: How Can It Be Supported? *Educational Action Research*, *13*(4), 525-542. <http://libproxy1.nus.edu.sg/login?url=https://search.proquest.com/docview/61887354?accountid=13876&bdid=6923&_bd=khCNxgu3JMbQ7rBFj%2BFiapaGKlE%3D>

Mezirow, J. (1990). *Fostering Critical Reflection in Adulthood: A Guide to Transformative and Emancipatory Learning*. Jossey-Bass.

Miller-Kuhlmann, R., O'Sullivan, P. S., & Aronson, L. (2016). Essential steps in developing best practices to assess reflective skill: A comparison of two rubrics. [References]. (1), 75-81.

Moniz, T., Arntfield, S., Miller, K., Lingard, L., Watling, C., & Regehr, G. (2015). Considerations in the use of reflective writing for student assessment: Issues of reliability and validity. [References]. (9), 901-908.

Murdoch-Eaton, D., & Sandars, J. (2014). Reflection: moving from a mandatory ritual to meaningful professional development. *Arch Dis Child*, *99*(3), 279-283. <https://doi.org/10.1136/archdischild-2013-303948>

Naeger, D. M., Hua, E. W., Ahearn, B., & Webb, E. M. (2015). Reflective Writing: A Potential Tool to Improve Interprofessional Teamwork with Radiologists. *Acad Radiol*, *22*(10), 1221-1225. <https://doi.org/10.1016/j.acra.2015.07.006>

Nagano, H., Obara, H., & Takayama, Y. (2019). A brief home-based palliative care learning experience for medical students and resident doctors in Okinawa, Japan. *PLOS ONE*, *14*(6).

Nguyen, Q. D., Fernandez, N., Karsenti, T., & Charlin, B. (2014). What is reflection? A conceptual analysis of major definitions and a proposal of a five-component model. *Medical education*, *48*(12), 1176-1189.

Olex, A. L., DiazGranados, D., McInnes, B. T., & Goldberg, S. (2020). Local Topic Mining for Reflective Medical Writing. *AMIA Summits on Translational Science Proceedings*, *2020*, 459.

Pavlovich, K. (2007). The development of reflective practice through student journals. *Higher Education Research and Development*, *26*(3), 281-295.

Pavlovich, K., Collins, E., & Jones, G. (2009). Developing students' skills in reflective practice: Design and assessment. *Journal of Management Education*, *33*(1), 37-58.

Rees, C. E., Shepherd, M., & Chamberlain, S. (2005). The utility of reflective portfolios as a method of assessing first year medical students' personal and professional development. *Reflective Practice*, *6*(1), 3-14.

Roberts, P. (2016). Reflection: A Renewed and Practical Focus for an Existing Problem in Teacher Education. *Australian Journal of Teacher Education*, *41*(7), 19-35. <http://libproxy1.nus.edu.sg/login?url=https://search.proquest.com/docview/1871574864?accountid=13876&bdid=6923&_bd=d1XiDYEoDKQyNxZUB5Jc9cgXR6g%3D>

Rosenbaum, M. E., Lobas, J., & Ferguson, K. (2005). Using reflection activities to enhance teaching about end-of-life care. *J Palliat Med*, *8*(6), 1186-1195. <https://doi.org/10.1089/jpm.2005.8.1186>

Ryan, M., & Ryan, M. (2013). Theorising a Model for Teaching and Assessing Reflective Learning in Higher Education. *Higher Education Research and Development*, *32*(2), 244-257. <http://libproxy1.nus.edu.sg/login?url=https://search.proquest.com/docview/1509088057?accountid=13876&bdid=6923&_bd=qOXg1yL4Htn9aqLd4EZSLZexA0I%3D>

Shapiro, J., Kasman, D., & Shafer, A. (2006). Words and wards: a model of reflective writing and its uses in medical education. *J Med Humanit*, *27*(4), 231-244. <https://doi.org/10.1007/s10912-006-9020-y>

Shaughnessy, A. F., Allen, L., & Duggan, A. (2017). Attention without intention: explicit processing and implicit goal-setting in family medicine residents' written reflections. *Educ Prim Care*, *28*(3), 150-156. <https://doi.org/10.1080/14739879.2016.1278562>

Standal, Ø., & Rugseth, G. (2014). Practicum in adapted physical activity: a Dewey-inspired action research project. *Adapt Phys Activ Q*, *31*(3), 219-239. <https://doi.org/10.1123/apaq.2013-0105>

Sukhato, K., Sumrithe, S., Wongrathanandha, C., Hathirat, S., Leelapattana, W., & Dellow, A. (2016). To be or not to be a facilitator of reflective learning for medical students? a case study of medical teachers' perceptions of introducing a reflective writing exercise to an undergraduate curriculum. *BMC Med Educ*, *16*, 102. <https://doi.org/10.1186/s12909-016-0624-2>

Sweet, L., Bass, J., Sidebotham, M., Fenwick, J., & Graham, K. (2019). Developing reflective capacities in midwifery students: Enhancing learning through reflective writing. *Women Birth*, *32*(2), 119-126. <https://doi.org/10.1016/j.wombi.2018.06.004>

Tawanwongsri, W., & Phenwan, T. (2019). Reflective and feedback performances on Thai medical students' patient history-taking skills. *BMC Med Educ*, *19*(1), 141. <https://doi.org/10.1186/s12909-019-1585-z>

Thorpe, K. (2004). Reflective learning journals: From concept to practice. *Reflective Practice*, *5*(3), 327-343.

Too, W. K. (2013). Facilitating the Development of Pre-Service Teachers as Reflective Learners: A Malaysian Experience. *Language Learning Journal*, *41*(2), 161-174. <http://libproxy1.nus.edu.sg/login?url=https://search.proquest.com/docview/1509080311?accountid=13876&bdid=6923&_bd=7vDQmEgYOCnwsqz4eF9JBABW5m4%3D>

Tsingos, C., Bosnic-Anticevich, S., Lonie, J. M., & Smith, L. (2015). A Model for Assessing Reflective Practices in Pharmacy Education. *Am J Pharm Educ*, *79*(8), 124. <https://doi.org/10.5688/ajpe798124>

Tsingos, C., Bosnic-Anticevich, S., & Smith, L. (2014). Reflective practice and its implications for pharmacy education. *Am J Pharm Educ*, *78*(1), 18. <https://doi.org/10.5688/ajpe78118>

Vettraino, E., Linds, W., & Downie, H. (2019). Embodied reflexivity: Discerning ethical practice through the Six-Part Story Method. *Reflective Practice*, *20*(2), 218-233.

Vicini, A., Shaughnessy, A. F., & Duggan, A. P. (2017). Cultivating the Inner Life of a Physician Through Written Reflection. *Ann Fam Med*, *15*(4), 379-381. <https://doi.org/10.1370/afm.2091>

Wald, H. S., Reis, S. P., Monroe, A. D., & Borkan, J. M. (2010). 'The Loss of My Elderly Patient:' Interactive reflective writing to support medical students' rites of passage. *Med Teach*, *32*(4), e178-184. <https://doi.org/10.3109/01421591003657477>

Wang, Y.-H., & Liao, H.-C. (2020). Construction and Validation of an Analytic Reflective Writing Scoring Rubric for Healthcare Students and Providers. *醫學教育*, *24*(2), 53-72.

Wear, D., Zarconi, J., Garden, R., & Jones, T. (2012). Reflection in/and writing: pedagogy and practice in medical education. *Acad Med*, *87*(5), 603-609. <https://doi.org/10.1097/ACM.0b013e31824d22e9>

Winkel, A. F., Yingling, S., Jones, A. A., & Nicholson, J. (2017). Reflection as a Learning Tool in Graduate Medical Education: A Systematic Review. *J Grad Med Educ*, *9*(4), 430-439. <https://doi.org/10.4300/jgme-d-16-00500.1>

Wong, K. Y., Loke, A. Y., Wong, M., Tse, H., Kan, E. & Kember, D. (1997). An action research study into the development of nurses as reflective practitioners. *Journal of Nursing Education*, *34*(10), 476-481.

Xiao, Q., Zhu, P., Hsu, M. K., Zhuang, W., & Peltier, J. (2016). Reflective Learning in a Chinese MBA Programme: Scale Assessment and Future Recommendations. *Journal of Further and Higher Education*, *40*(1), 1-22. <http://libproxy1.nus.edu.sg/login?url=https://search.proquest.com/docview/1773231055?accountid=13876&bdid=6923&_bd=DE9Pv%2BTJhX7X8s5p0bdO9ccKp8s%3D>

Zoellner, B. P., Chant, R. H., & Lee, K. (2017). Do We Do Dewey? Using a Dispositional Framework to Examine Reflection within Internship Professional Development Plans. *Teacher Educator*, *52*(3), 203-221. <http://libproxy1.nus.edu.sg/login?url=https://search.proquest.com/docview/1969019113?accountid=13876&bdid=6923&_bd=ZjE%2BhiVPETDbwn6fcXSq0V55TPM%3D>
